# Exploring the psychometric properties of the professional issues in maternal mental health scale (PIMMHS) in a Chinese population

**DOI:** 10.1186/s12955-023-02106-0

**Published:** 2023-03-27

**Authors:** Lu Lin, Huimin Guo, Qian Fang, Colin R. Martin, Aiying Jin, Congyan Xie, Li Tian, Julie Jomeen

**Affiliations:** 1 The First Affiliated Hospital of Soochow University, No. 188 Shizi Road, Suzhou, 215006 China; 2 School of Nursing, Medical College of Soochow University, Suzhou, China; 3 Institute of Health and Well-Being, University of Suffolk, Ipswich, UK; 4 Faculty of Health, Southern Cross University, Bilinga, Gold Coast, QLD 4225 Australia

**Keywords:** Perinatal, Mental health, Scale, Psychometric, Practitioner

## Abstract

**Background:**

The public health and economic implications of perinatal mental health problems are well documented. Maternity clinicians are ideally placed to effectively identify women at risk and facilitate early intervention. However, in China as globally a number of issues are implicated in a failure to recognise and treat.

**Aim:**

The present study sought to develop and evaluate the Chinese version ‘professional issues in maternal mental health’ scale (PIMMHS), explore its psychometric properties and potential application.

**Methods:**

A cross-sectional design and instrument translation and evaluation approach was taken to investigate the psychometric properties of the PIMMHS in a Chinese population. A total of 598 obstetricians, obstetric nurses, and midwives participated in this study from 26 hospitals across China.

**Findings:**

The Chinese PIMMHS was not a good fit to the original two factor model. The emotion/communication subscale yielded an excellent fit to the data according to all fit indices, offering compelling evidence for a single factor solution**.** The training (PIMMHS: Training), proved problematic throughout the analysis with divergent validity for the training subscale also being poor with a concomitant impact on the total scale performance. The performance of this subscale may be related to the nature of medical training and PMH.

**Conclusion:**

The Chinese PIMMHS comprises a unidimensional scale of emotion/ communication, which is simple and may provide insight into the emotional burden of providing PMH care, with the potential to mitigate that burden. Further development and investigation of the training sub-scale could be of value.

## Introduction

Perinatal mental health (PMH) is a globally recognised public health problem [1], referring to women’s mental health from pregnancy to the first year after birth [2].Perinatal mental health problems (PMHP) refer to the range of mental disorders that women may encounter during this period, from anxiety and depression to more serious mental diseases [3].The consequences of PMHP in terms of deleterious outcomes for maternal, paternal, child and societal outcomes is internationally documented [4]. Women suffering from PMHP have lower self-esteem, higher levels of anger, poor interpersonal relationship, and higher suicidal and infanticide tendency [5–9]. Impacts on fetal health and child development in the short and long term have also been demonstrated [10].

In China, the prevalence of perinatal depression is 17.4% and is on the rise [11], and the prevalence of perinatal anxiety ranges from 7.45 to 38.6% [11–13]. In addition, several studies illustrate a prevalence of post-partum post-traumatic stress ranging from 1.13 to 11.38% [14–16]. The burden of PMHP in individual, societal, and economic terms has now been evidenced and must not be underestimated [17]. In recent years, the introduction of the two-child policy in China has potential consequences for the incidence of PMHP in the childbearing population, which calls for the expansion of healthcare services for mothers and infants.

The perinatal period is a time of high healthcare utilisation [18], and hence an opportunistic period for the identification of PMHP. Practitioners caring for women during this period are in an ideal position to effectively identify women at risk and facilitate early intervention [19]. However, numerous factors have been identified as problematic, which for the Chinese population include stigma and a reluctance of women to disclose [20]. Cultural issues come into play with some cultures uncomfortable with the construct of mental health, creating difficulties for practitioners in managing PMHP [21]. Failure or reluctance by healthcare practitioners to recognise of the signs of PMHPs linked to both a lack of skills and/or resources is pertinent in China with both health professionals and the public lacking knowledge of mental health problems [20].

The context of maternity settings, the time limited nature of consultations, as lack of referral options and the absence of care pathways have been identified as barriers to the identification and effective management of PMHP [4, 22] and indeed have been cited as problematic in the Chinese context [20]. Women therefore, are a risk of not seeking and/or receiving adequate professional help, particularly during the perinatal period.

To understand contextual organization and support barriers to delivering PMH care, service providers require insight into the key professional issues. The following paper describes an assessment tool that identifies areas of practice, which create challenge for practitioners, to support focused service development and training. It also offers the opportunity to evaluate any changes made in supporting practitioners to optimize their role in PMH.

To date this tool has been validated in a UK and an Irish population. It is acknowledged that whilst many similar issues have been identified across international contexts in relation to PMH care, cultural context may be relevant to the utility of any assessment tool.

The goal of the current investigation was to evaluate the measurement properties of the Chinese version of the PIMMHS.

Using a design similar to the original instrument development and validation study [23], the study research questions were:

i. Does the PIMMHS scale comprise two correlated sub-scales of (i) emotion and (ii) training consistent with the original PIMMHS bi-dimensional measurement model?
ii. Do the PIMMHS total score and associated sub-scales demonstrate acceptable internal consistency?
iii. Do the PIMMHS total measure and identified sub-scales demonstrate acceptable divergent reliability?
iv. Do the PIMMHS total measure and identified sub-scales demonstrate acceptable convergent reliability?
v. Do the PIMMHS total measure and identified sub-scales demonstrate acceptable known-groups discriminant validity?

## Methods

### Design and participants

A cross-sectional design was used that incorporated an embedded between-groups component of professional group type (i. obstetrician, ii. maternity nurse, iii. Midwife). Following the approach of Jomeen et al. [23] a standard battery of validity and reliability statistical tests were used to evaluate the Chinese version of the PIMMHS [17, 24].

### Participants

The study was approved by the Ethics Committee of Soochow University (Approval No. SUDA20200225H09). All procedures performed in studies involving human participants were in accordance with the ethical standards of the institutional and/or national research committee and with the 1964 Helsinki declaration and its later amendments or comparable ethical standards. In December 2019, data were collected using Sojump.com (Questionnaire Star, which is a professional online questionnaire survey, evaluation and voting platform) at the Obstetrics and Gynecology Department of 26 hospitals in Suzhou, Nanjing, Changshu, and Yangzhou in China. A total of 598 obstetricians, obstetric nurses, and midwives participated in this study. The inclusion criteria were: (i) Obstetricians, obstetric nurses, and midwives with professional qualification certificate; (ii) Informed consent and volunteer to participate in the study. The exclusion criteria were interns and trainees.

## Measures

### Professional issues in maternal mental health scale (PIMMHS)

The Professional issues in maternal mental health scale (PIMMHS) [23] is a short 7-item self-report measure designed to assess two related domains of (i) emotional burden in relation to health practitioner’s current knowledge and skills base and, (ii) perception of training skills base adequacy in relation to working with and engaging with individuals with potential perinatal mental problems and perinatal mental health issues more generally. The exploratory factor analysis revealed the PIMMHS had excellent model fit: *χ*^2^_(df=8)_ = 9.70, CFI = 0.99, RMSEA = 0.03, RMSR = 0.02, df-corrected RMSR = 0.04[23]. The Cronbach’s alpha coefficients of the PIMMHS-Emotion sub-scale and the PIMMHS-Training sub-scale were 0.91 and 0.57 respectively [23].

### Multi-dimensional health locus of control (MHLC) scale

Locus of control was assessed by an adapted version of Form C of the Multi-dimensional Health Locus of Control (MHLC) scale developed by Wallston and colleagues [25]. There were four sub-scales: Internal, Chance, Doctors and Others in the MHLC scale. The confirmatory factor analysis revealed the Chinese version of MHLC Scale had good model fit: χ^2^_(df=129)_ = 357.40, RMSEA = 0.06, CFI = 0.73, Tucker–Lewis index (TLI) = 0.68[26]. The Chinese version of the three-item MHLC ‘Doctors’ sub-scale was used in the current study with its Cronbach alpha coefficient being 0.51 [26].

### Perinatal mental health awareness (PMHA) scale

The Perinatal Mental Health Awareness (PMHA) [17] is a brief 9-item scale designed to measure awareness of (i) knowledge, (ii) identification and, (iii) management of perinatal mental health problems and issues. The PMHA scale comprises three sub-scale of stress, anxiety, and depression, learning difficulty and physical/medical issues) of three questions each. The exploratory factor analysis revealed the PMHA scale had good model fit: χ^2^_(df=12)_ = 36.77, CFI = 0.96, RMSEA = 0.09, RMSR = 0.03, df-corrected RMSR = 0.05. The Cronbach’s alpha coefficients of the PMHA total scale, the PMHA-SAD, PMHA-MED and PMHA-LD sub-scales were 0.79, 0.68, 0.77 and 0.78 respectively.

### The perinatal illness perceptions scale (PIPS)

The perinatal illness perceptions scale (PIPS) [27] is a psychometrically robust measure of health practitioners’ perceptions of perinatal mental health. There were three domains in this scale: PIPS-Causes, PIPS-Mother and PIPS-Baby. The exploratory factor analysis revealed the PIPS scale had good model fit: χ^2^_(df=250)_ = 537.34, CFI = 0.87, RMSEA = 0.07, RMSR = 0.05, df-corrected RMSR = 0.06. The PIPS ‘Causes’ sub-scale was used in the context of the current study, and its Cronbach’s alpha coefficient was 0.90.

## Translation

**Fig. 1:**
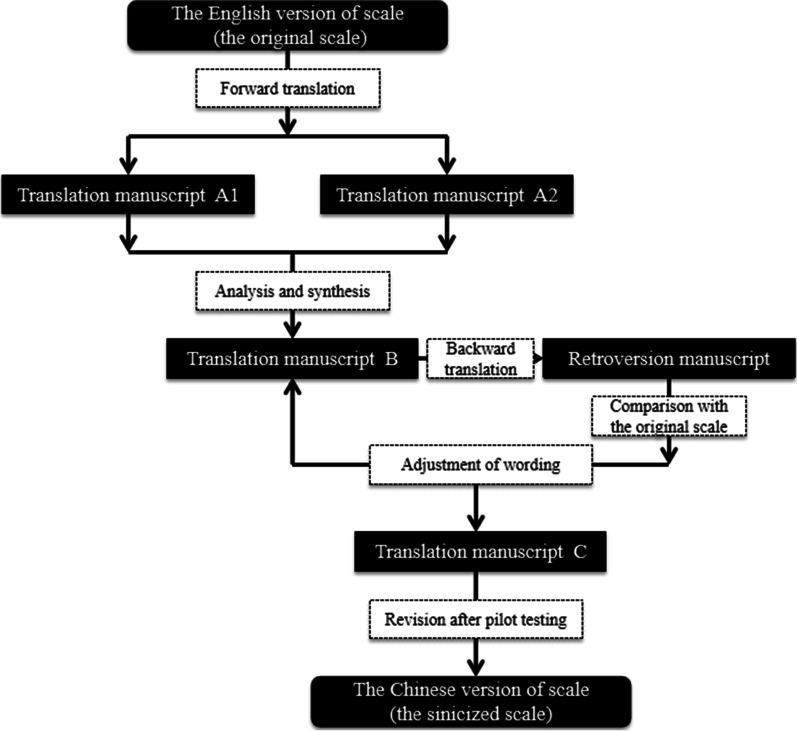
Sinicization flow chart of the PIMMHS/MHLC/PMHA/PIPS scale

These four scales were translated from English into Chinese using Brislin’s translation model [28]. The steps for sinicization of these scales are shown in Fig. 1. Firstly, two bilingual researchers separately translated the original PIMMHS/MHLC/PMHA/PIPS scale into Chinese. After the discrepancies between these two translations were reviewed and discussed comprehensively, a single version was formed, which was then translated back into English by another bilingual researcher. The retroversion was repeatedly compared with the original PIMMHS/MHLC/PMHA/PIPS scale and the Chinese expressions were adjusted accordingly. During this procedure, the translation validity index (TVI) was used to assess the translation equivalence of different versions. A 4-point Likert scale (1 = uncorrected, 2 = needs major modification on equivalent item, 3 = equivalent but needs minor modification, and 4 = equivalent) was used. In this study, three language experts were recruited to compare the scale in English and Chinese. The items were revised until a TVI score of 4 was achieved. The revised version of the scale was pilot tested with a convenience sample of 30 midwives in the First Affiliated Hospital of Soochow University to evaluate whether the Chinese version of the scale was easy to understand. Language expressions were adjusted if midwives felt the wording was difficult to understand. After the pilot test, the Chinese version of all scales were finalised for the test of its psychometric properties.

## Statistical analysis

### Construct validity

Construct validity of the scale was tested by Confirmatory Factor Analysis (CFA) using Amos software at α = 0.05. The fitness of the model was evaluated by the chi-squared freedom ratio (χ^2^/df), Root Mean Square Error of Approximation (RMSEA), Comparative Fit Index (CFI), Incremental Fit Index (IFI), and Root Mean-square Residual (RMR) [29–33]. The collected samples were randomly divided into three parts using simple randomization and used for CFA of each of the three subscales.

### Divergent validity

Divergent validity was evaluated by correlating PIMMHS sub-scale scores with the MHLC ‘doctors’ sub-scale score consistent with the divergent validity testing of Martin et al. [17]. No statistically significant relationship between PIMMHS sub-scales and the MHLC ‘doctors’ sub-scale score was predicted.

### Convergent validity

Convergent validity was evaluated by correlating PIMMHS total and sub-scale scores with the PMHA ‘Stress, Anxiety and Depression (SAD)’ sub-scale, a construct considered likely to be conceptually aligned to the PIMMHS [23]. Statistically significant positive correlations are predicted between PMHA total and sub-scale scores and the PIMMHS training sub-scale score.

### Known-groups discriminant validity

Known-groups discriminant validity was evaluated by comparing those categorised above or below the median on the PIPS ‘Causes’ sub-scale using the between-subject *t*-test. It was predicted that those who scored above the median on the PIPS ‘Causes’ sub-scale would have significantly higher PIMMHS scores across all sub-scales and the total PIMMHS score.

### Reliability tests

In this study, internal consistency and test–retest reliability (reliability coefficient) were used for reliability testing. The former was derived by calculating the Cronbach’s alpha coefficients of the total scale and the two sub-scales and the latter by calculating Pearson’s correlation coefficient. A Cronbach’s alpha of 0.70 or greater is indicative of acceptable internal reliability [24, 34]. Statistical analysis was undertaken using the statistical software package R [35].

## Descriptive results

### Results

Five-hundred and ninety-eight participants took part in the study, roughly equally distributed among obstetricians (N = 198), maternity nurses (N = 200) and midwives (N = 200). Evaluation of Mahalanobis distances revealed the presence of 15 multivariate outliers in the dataset and these participants were consequently excluded from further analysis (final dataset N = 588, obstetricians N = 192 (33%), maternity nurses N = 197 (34%), midwives N = 199 (34%). The means, standard deviations, skew, and kurtosis of each PIMMHS item are shown in Table 1 below. Skew and kurtosis characteristics for each item indicate a univariate normal distribution (skew < 3, kurtosis < 10). The mean and standard deviations of the PIMMHS total score and PIMMHS-Emotion and PIMMHS-Training sub-scales were 16.78 (3.92), 9.42 (2.80) and 7.36 (1.61) respectively.

**Table 1:** Individual item and distributional characteristics of the professional issues in maternal mental health scale (PIMMHS)

PIMMHS item	Sub-scale	Mean	SD	Skew	Kurtosis	SE.k
PIMMHS 1	Emotion	2.83	0.73	− 0.38	0.06	0.03
PIMMHS 2	Emotion	2.14	0.98	− 0.01	− 0.97	0.04
PIMMHS 3	Emotion	2.19	0.98	− 0.12	− 0.88	0.04
PIMMHS 4	Emotion	2.26	0.95	− 0.19	− 0.86	0.04
PIMMHS 5	Training	2.11	0.95	− 0.01	− 1.01	0.04
PIMMHS 6	Training	2.98	0.74	− 0.50	0.21	0.03
PIMMHS 7	Training	2.26	0.82	− 0.10	− 0.50	0.03

*SE.k* Standard error of kurtosis

### Group comparisons

One-way between groups analysis of variance (ANOVA) revealed highly statistically significant differences on PIMMHS total and PIMMHS-training sub-scale scores based on professional affiliation as the grouping variable (Table 2.). Post-hoc Bonferroni-adjusted comparisons revealed statistically significant differences between maternity nurses and obstetricians (*p* < 0.05) and between midwives and obstetricians (*p* < 0.05) with the nurses and midwives having higher PIMMHS total and PIMMHS-Training sub-scale scores.

**Table 2:** One-way between-subjects ANOVA of PIMMHS sub-scale and total scores as a function of group type

PIMMHS scale	MN	MW	OB	Degrees of freedom		Sums of mean		F	*p*
	Mean (SD)					Squares	Square		
PIMMHS emotion	9.66 (2.88)	9.47 (2.97)	9.13 (2.50)	Group	2	27.80	13.90	1.78	0.17
				Residuals	585	4567.90	7.81		
PIMMHS training	7.49 (1.59)	7.62 (1.71)	6.95 (1.45)	Group	2	49.57	24.78	9.79	< 0.001
				Residuals	585	1481.43	2.53		
PIMMHS total	17.15 (3.98)	17.09 (4.16)	16.08 (3.52)	Group	2	139.70	69.83	4.60	0.01
				Residuals	585	8890.50	15.20		

*MN* Maternity nurses, *MW* Midwife, *OB* Obstetrician, *SD* Standard deviations

### Construct validity

358 cases were used for the CFA of the Chinese Version of Perinatal Illness Perceptions Scale (PIPS-C), 135 cases for the Chinese Version of Perinatal Mental Health Awareness (PMHA-C), and 105 cases for the Chinese Version of professional issues in maternal mental health scale (PIMMHS-C).

**Fig. 2:**
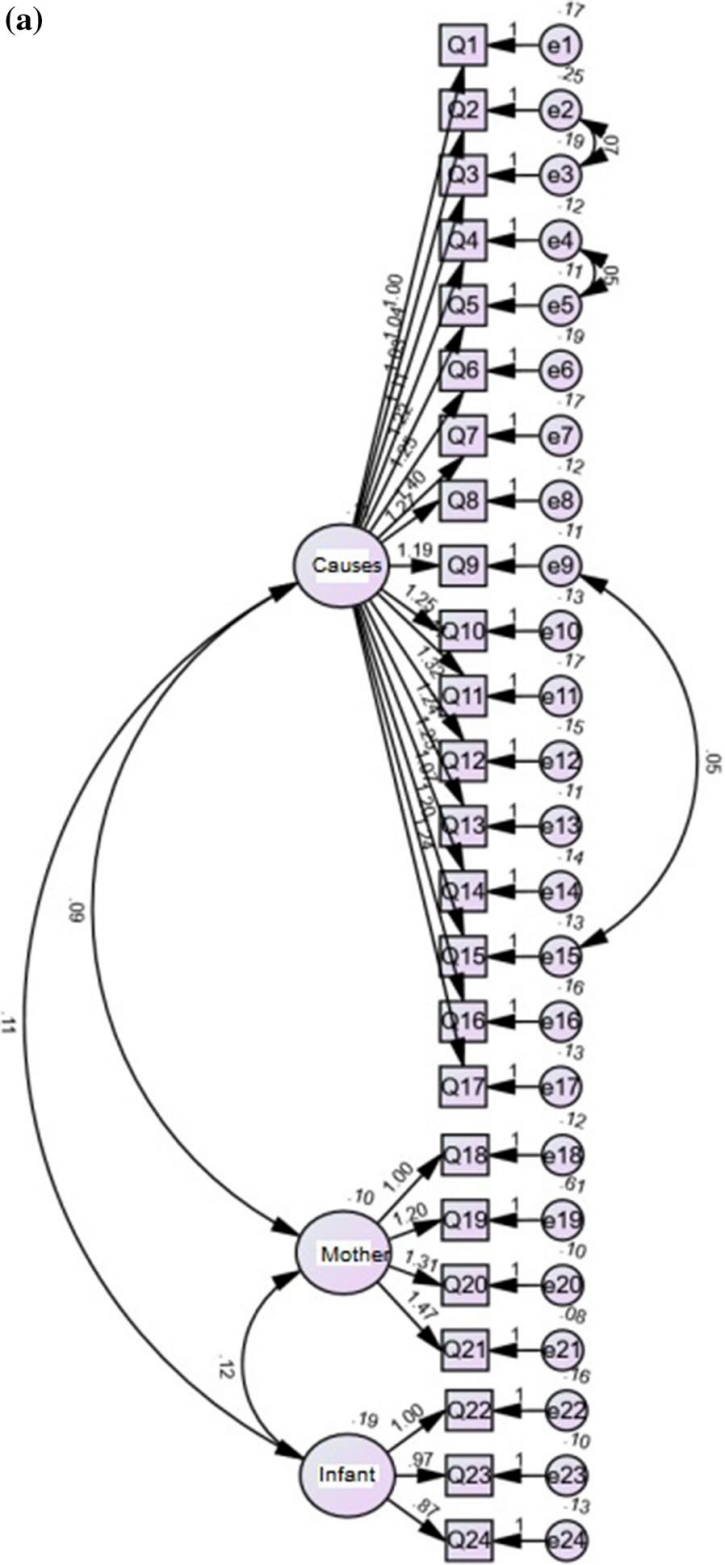

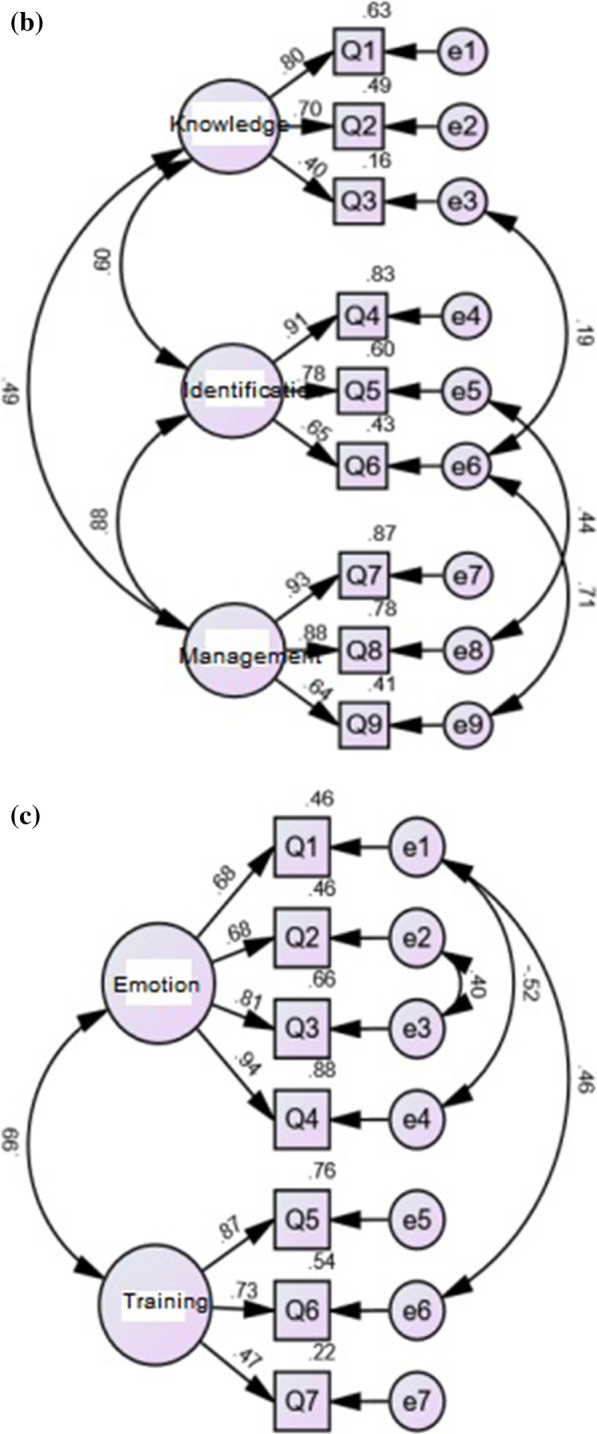
Results of the confirmatory factor analysis for the modified **a** PIPS-C (n = 358), **b** PMHA-C (n = 135), **c** PIMMHS-C (n = 105)

A roadmap was drawn according to the 3-factor model of the original PIPS-C scale and the constructed model was tested using the maximum likelihood method. Model correction was applied to the initial model based on the MI (Modification Indices). 3 residuals Cov (e2, e3), Cov (e4, e5), and Cov (e9, e15) were set as free parameters to improve the model fit. The modified model fit indices were χ^2^/df = 3.241, RMSEA = 0.079, CFI = 0.901, IFI = 0.902 and RMR = 0.017. The modified model is shown in Fig. 2a and the model fit results are presented in Table 3.A 3-factor a priori model following the original PMHA-C scale was used as the latent variable to plot the pathway and the constructed model was tested using the maximum likelihood method. Model correction was applied to the initial model based on the MI (Modification Indices). 3 residuals Cov (e3, e6), Cov (e5, e8), and Cov (e6, e9) were set as free parameters to improve the model fit. The modified model fit indices were χ^2^/df = 2.289, RMSEA = 0.098, CFI = 0.964, IFI = 0.965 and RMR = 0.018. The modified model is shown in Fig. 2b and the model fit results are shown in Table 3.

**Table 3:** Confirmatory factor analysis models

Model	Chi square (df)	CFI	RMSEA	SRMR
Single-factor (all items)	288.73 (14)	0.86	0.18	0.116
Single-factor (Emotion items)	2.03 (2)	0.99	0.005	0.009

*CFI* Comparative fit index, *RMSEA* Root mean error of approximation, *SRMR* Squared root mean residual

A 2-factor a priori model following the original PIMMHS-C scale was used as the latent variable to plot the pathway and the constructed model was tested using the maximum likelihood method. Model correction was applied to the initial model based on the MI (Modification Indices). 3 residuals Cov (e1, e6), Cov (e1, e4), and Cov (e2, e3) were set as free parameters to improve the model fit. The modified model fit indices were χ^2^/df = 3.135, RMSEA = 0.143, CFI = 0.956, IFI = 0.957 and RMR = 0.038. The modified model is shown in Fig. 2c and the model fit results are shown in Table 3.

### Divergent validity

Correlations between PIMMHS total and sub-scale scores and the MHLC ‘Doctors’ sub-scale are summarised in Table 4. Against prediction, correlations between the PIMMHS total score and PIMMHS-Training sub-scale were noted to be statistically significant (*p* < 0.05).

**Table 4:** Pearson’s *r* correlation coefficients between PIMMHS sub-scale and total score and the MHLC ‘Doctors’ sub-scale (divergent validity) and PMHA-SAD subscale (convergent validity)

Scale	Scale				
	PIMMHS emotion	PIMMHS training	PIMMHS total	MHLC-‘doctors’	PMHA-SAD
PIMMHS emotion		0.55*	0.94*	0.06	0.22*
PIMMHS training			0.80*	0.27*	0.42*
PIMMHS total				0.15*	0.33*

*p < 0.05

### Convergent validity

Significant positive correlations were observed between all PIMMHS total and sub-scale scores and the PMHA-SAD sub-scale (PIMMHS total, *r* < 0.33, *p* < 0.001; PIMMHS-Emotion sub-scale, *r* = 0.22, *p* < 0.001, PIMMHS-Training sub-scale, *r* = 0.42, *p* < 0.001).

### Known-groups discriminant validity

Statistically significant differences were observed for both PIMMHS sub-scales and the.

PIMMHS total score as a function of the median-split PIPS-Causes sub-scale classification.

(Table 5). Cohen’s *d* revealed effect sizes to be small for all between-groups comparisons.

**Table 5:** Medium-split group categorisation on the PIPS-Causes sub-scale

Variable	AM (n = 295)	BM (n = 293)	t	*df*	*p*	Cohen’s *d*	*d* 95% CI	Effect size
	Mean (SD)							
PIMMHS-total	17.43 (4.25)	16.13 (3.45)	4.07	586	< 0.001	0.34	0.17–0.50	Small
PIMMHS-emotion	9.75 (3.00)	9.10 (2.55)	2.83	586	0.005	0.23	0.07–0.40	Small
PIMMHS-training	7.68 (1.75)	7.03 (1.39)	4.98	586	< 0.001	0.41	0.25–0.57	Small

*AM* Above median, *BM* Below median, *SD* Standard deviations

### Internal consistency

Cronbach’s alpha of the PIMMHS total scale, PIMMHS-Emotion and PIMMHS-Training sub-scales were 0.852, 0.784 and 0.682 respectively (Table 6).

**Table 6:** Reliability test results (n = 598)

Dimension	Number of items	Cronbach’s α coefficient	Test–retest reliability
PIPS-C	24	0.959	0.944
PIPS-C-causes	17	0.954	0.867
PIPS-C-mother	3	0.726	0.832
PIPS-C-infant	4	0.795	0.815
PMHA-C	9	0.908	0.945
PMHA-C-knowledge	3	0.698	0.912
PMHA-C-identification	3	0.844	0.907
PMHA-C-management	3	0.856	0.877
PIMMHS-C	7	0.852	0.879
PIMMHS-C-emotion	4	0.784	0.851
PIMMHS-C-training	3	0.682	0.715

## Discussion

The current investigation sought to determine if the PIMMHS items could be translated to a Chinese context and retain it psychometric properties, providing an effective measure for assessing professional issues in the context of providing PMH care in the clinical. The descriptive review of the individual item distributional characteristics suggested, in terms of a robust psychometric appraisal of measurement qualities, underpinned the suitability of using a statistical approach underpinned by parametric assumptions of data normality.

CFA using the factor structure outlined in the original validation paper [23] revealed a lack of fit to the two-factor model. The singular cause of this appears to be the training subscale, contrary to the original UK and subsequent Irish validation study. It was noteworthy that the consequent single factor solution, comprising the emotion sub-scale did yield an excellent fit to the data according to all fit indices, offering compelling evidence for a single factor solution, with a good Cronbach alpha and thus, extrapolating from this, that the Chinese PIMMHS can only be utilised as a unidimensional measure assessing the emotional burden of managing PMHPs in practice.

The training score proved problematic throughout the analysis with divergent validity for the training subscale also being poor with a concomitant impact on the total scale performance. However, the divergent validity was good for the emotion sub-scale, as was convergent and known groups discriminant validity. This potentially offers important insight into the sensitivity of the emotion sub-scale to the relationship between knowledge and confidence in managing PMH and emotional burden. This is fundamentally important as indicates the value of PMH knowledge, confidence, and appropriate illness perceptions in relation to clinical behaviour and engagement with women with PMHP.

It is of interest here to speculate on the content of the training questions and why they performed so badly. These questions were all focused on the value of training to equip practitioners to deliver PMH care but derived broadly from Western literature and practice. It might be feasible to suggest that these questions have less relevance in China or are influenced by the context of training in the Chinese setting. Whilst health care professionals in China consider that PMH is an important issue, they identify structural issues in service provision and a legacy of the perception of mental health as unimportant [20], which in turn is likely to have been reflected in health curricula and practitioner preparation to deal with PMHPs. It is however of further interest within this speculation to note that the obstetricians revealed much lower scores than the nurses or the midwives potentially reflecting the training obstetricians receive in terms of PMH or a context in which PMH is not seen as an obstetric concern.

Further enquiry would be extremely valuable in determining whether the PIMMHS-training sub-scale could be revised for the Chinese context, However, it could also be suggested that the training subscale is culturally mediated and whether the functionality of this sub-scale can be replicated across different cultural contexts is a question that must be asked.

The study was not without limitations, which could be addressed in future work on the measure. The survey design approach did not allow the opportunity to evaluate test–retest reliability, which further work should seek to do, particularly if looking at the PIMMHS Emotion as a unidimensional measure, using a 12-week pre-post repeated-measures design consistent with the recommendations of Kline [22]. The sensitivity of the PIMMHS Emotion sub-scales to intervention would be useful to fully determine the value of the measure.

## Conclusion

The PIMMHS did not perform in line with the original UK study, with the training sub-scale and hence the total scale performing badly. However, the emotion sub-scale did perform well and perhaps offers some utility in the Chinese context. It is simple and easy to use and may provide insight into the emotional burden of providing PMH care, with the potential to mitigate that burden by enhancing maternity practitioner’s knowledge and confidence in this space. There are outstanding questions about the utility and replicability of the training sub-scale, which may be worthy of further consideration.

## Data Availability

All data generated or analysed during this study are included in this published article.
